# An Atypical Presentation of Lemmel's Syndrome: A Rare Culprit of Intermittent Biliary Obstruction

**DOI:** 10.1002/ccr3.71877

**Published:** 2026-02-03

**Authors:** Tinsae Anebo, Michael Negussie, Phuuwadith Wattanachayakul, Karecia Byfield, Hamza Tahir, Adrian Zhou, Ghideon Ezaz

**Affiliations:** ^1^ Department of Internal Medicine Jefferson Einstein Hospital Philadelphia Pennsylvania USA; ^2^ Department of Medicine Addis Ababa University Addis Ababa Ethiopia; ^3^ Department of Radiology Jefferson Einstein Hospital Philadelphia Pennsylvania USA; ^4^ Department of Gastroenterology and Hepatology Jefferson Einstein Hospital Philadelphia Pennsylvania USA

**Keywords:** biliary dilatation, biliary obstruction, extrahepatic biliary obstruction, Lemmel syndrome, periampullary diverticulum

## Abstract

Lemmel's syndrome can cause obstructive jaundice due to a periampullary duodenal diverticulum, leading to biliary obstruction without stones or tumors. The blockage can be intermittent as stasis in the diverticulum promotes enterolith or debris formation, which may temporarily obstruct the bile duct, causing fluctuating symptoms.

## Graphical abstract

Imaging features of Lemmel syndrome illustrating intermittent extrinsic compression of the common bile duct by a periampullary duodenal diverticulum. Current CT demonstrates abrupt CBD tapering with upstream biliary dilatation, while prior imaging shows preserved ductal caliber, supporting a dynamic obstructive process.

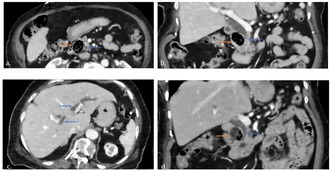

## 1 Introduction

Lemmel's syndrome is a rare condition where a periampullary diverticulum (PAD) compresses the distal common bile duct (CBD), causing obstructive jaundice in the absence of gallstones or malignancy [1]. PADs are pouch‐like protrusions of the duodenal mucosa located within 2–3 cm of the ampulla of Vater [2]. PADs are most often observed in older populations, with a prevalence as high as 27% [1]. Approximately 95% of PADs are asymptomatic, while 1%–5% of cases may result in Lemmel's syndrome [3]. Since its initial description by Dr. Gerhard Lemmel in 1934, a few case reports have documented this condition [4, 5, 6, 7, 8]. This report discusses a rare presentation of Lemmel's syndrome, emphasizing its clinical significance, diagnostic hurdles, and management strategies.

## 2 Case Presentation

### 2.1 History and Examination

A 90‐year‐old woman from the United States with a medical history that includes hypertension, hyperlipidemia, osteoporosis, gastroesophageal reflux disease (GERD), and diverticulosis previously complicated by bleeding presented to our hospital with a sudden onset of epigastric pain six‐hour duration. She described the pain as radiating from the epigastrium to both upper quadrants of her abdomen and noted several episodes of nausea and vomiting, which were non‐bloody and non‐bilious in nature. Upon arrival at the hospital, her vital signs were as follows: blood pressure 181/81 mmHg, heart rate 75 bpm, and she was afebrile. Despite her stable vital signs, she appeared to be in mild distress due to her ongoing pain. Physical examination revealed notable tenderness in the epigastric region, and there were no signs of scleral icterus or jaundice.

### 2.2 Investigations

**FIGURE 1:**
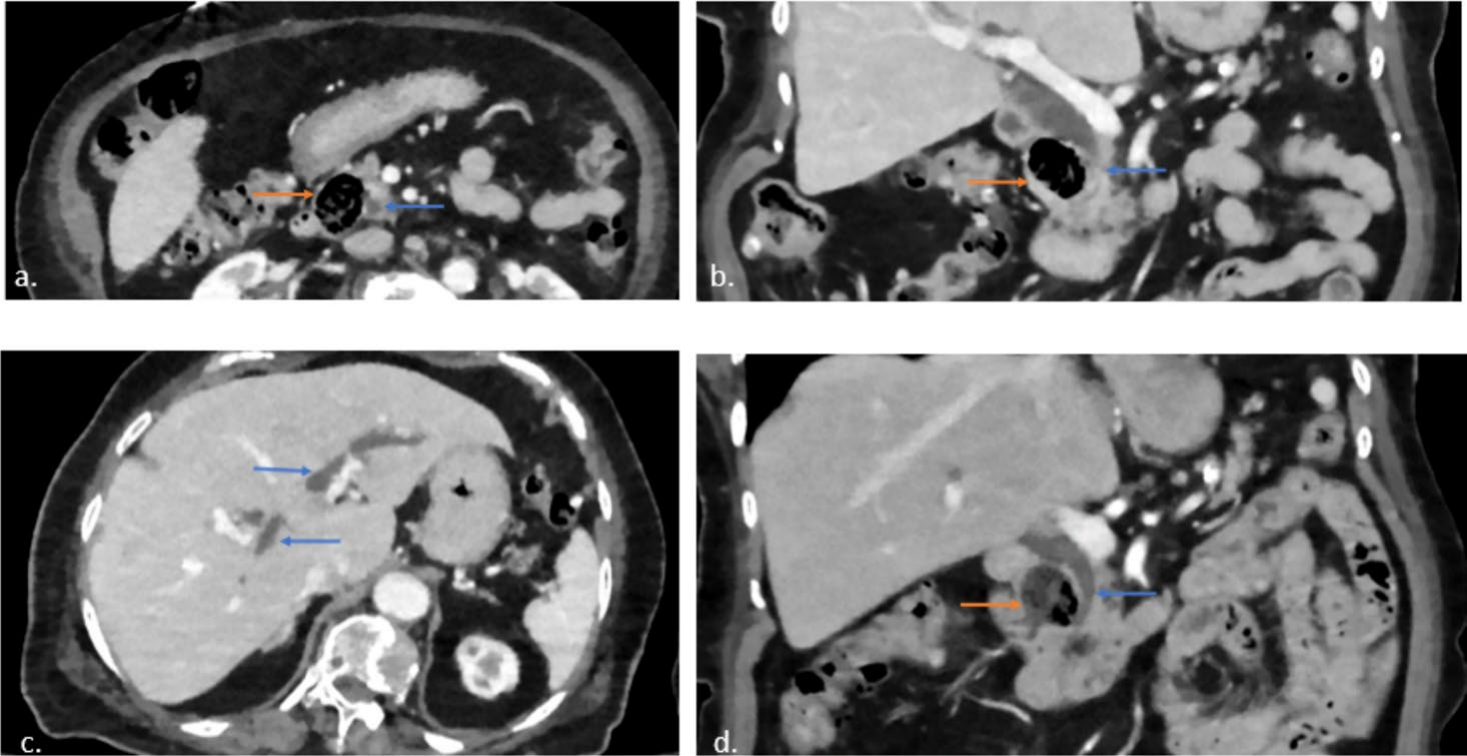
Lemmel syndrome: (a) Axial CT demonstrates prominent periampullary duodenal diverticulum (orange arrow) and adjacent common bile duct (CBD, blue arrow). (b) Coronal CT demonstrates abrupt tapering of the CBD (blue arrow), likely due to compression by the diverticulum (orange arrow). (c) There is diffuse upstream biliary ductal dilatation (blue arrow). (d) Prior CT from one year ago showed similar‐sized diverticulum (orange arrow) but smooth tapering of CBD (blue arrow) without evidence of intrahepatic biliary dilation (not shown). Therefore, intermittent obstruction of CBD by the diverticulum is suspected.

Initial laboratory tests showed a hemoglobin level of 12.9 g/dL, with normal white blood cell and platelet counts, as well as normal renal function and electrolyte levels. However, her liver function tests showed a total bilirubin of 25.7 μmol/L (direct fraction 10.3 μmol/L), alkaline phosphatase of 389 U/L, AST of 501 IU/L, and ALT of 281 IU/L. Her lipase level was within the normal range at 28 IU/L. An abdominal CT‐(Figure 1) revealed diffuse intra‐ and extrahepatic biliary ductal dilation. The common bile duct measured 1.7 cm proximally and tapered to 0.7 cm distally, where a 2.5 cm periampullary duodenal diverticulum appeared to be compressing the distal common bile duct. A comparison with a CT scan‐(Figure 1) conducted a year earlier showed a similar diverticulum, but only mild biliary ductal dilation at that time, and the patient had been asymptomatic with normal liver enzyme levels. These new findings raised concern for Lemmel syndrome, a rare condition in which a periampullary diverticulum intermittently obstructs the biliary tract, leading to cholestasis and explaining her acute presentation.

### 2.3 Outcome

Throughout her hospitalization, the patient was managed conservatively with pain relief, bowel rest and close monitoring. Her laboratory abnormalities, including the cholestatic enzyme levels, gradually improved without the need for any invasive interventions such as endoscopic procedures or surgery. By the time of discharge, her symptoms had significantly improved, and she reported substantial relief from her abdominal pain.

## 3 Discussion

The leading causes of obstructive jaundice are choledocholithiasis and tumors involving the pancreato‐biliary system or the periampullary region [9]. However, Lemmel's syndrome, a rare condition characterized by biliary duct compression due to a periampullary diverticulum (PAD), is also a recognized cause [10].

The development of Lemmel's syndrome due to PADs can be attributed to several mechanisms. First, PADs, often containing an enterolith or bezoar, can directly compress the distal common bile duct or the ampulla of Vater [11]. Second, PADs can lead to dysfunction of the sphincter of Oddi, disrupting bile flow [12]. Lastly, chronic inflammation associated with diverticulitis may result in persistent papillitis and fibrosis of the ampulla, further contributing to bile duct obstruction [3].

Lemmel's syndrome can be classified into three types based on the position of the major duodenal papilla: type I occurs when the papilla is located inside the diverticulum, type II when it is at the edge of the diverticulum, and type III when it is situated near the diverticulum [13].

Patients with Lemmel syndrome typically present with symptoms of biliary obstruction, such as abdominal pain, jaundice, and less frequently, nausea or vomiting. Our case features a 90‐year‐old woman presenting with acute epigastric pain, nausea, and vomiting, which align with the syndrome's clinical profile. Most reported cases of Lemmel's syndrome occur in patients in their 60s to 80s [5]. Our patient, at 90 years of age, represents one of the oldest documented presentations in the literature. Laboratory findings often reveal a cholestatic pattern, including elevated alkaline phosphatase, bilirubin, and transaminases. In this patient, the cholestatic enzyme elevation was notable, with a total bilirubin of 25.7 μmol/L (direct fraction 10.3 μmol/L), alkaline phosphatase of 389 U/L, and transaminases elevated at 501 IU/L (AST) and 281 IU/L (ALT). Imaging studies confirmed the diagnosis, with an abdominal CT showing intra‐ and extrahepatic biliary dilation and a 2.5 cm PAD compressing the distal CBD [6]. Given the imaging findings, our patient most likely has Type III Lemmel's syndrome, characterized by the major duodenal papilla being situated near the diverticulum, with resultant extrinsic compression of the common bile duct. An additional unique aspect of our case is the availability of comparative imaging over time. While most reported cases describe cross‐sectional imaging at a single time point [3], our patient had a prior CT scan one year earlier, which demonstrated a similar‐sized diverticulum without biliary dilation, followed by a subsequent CT scan showing marked biliary dilatation during symptomatic presentation. This longitudinal comparison provides stronger evidence for intermittent, extrinsic compression of the common bile duct by the diverticulum, supporting Lemmel's original description of the syndrome as an intermittent and sometimes elusive cause of biliary obstruction.

The differential diagnosis for biliary obstruction in Lemmel's syndrome includes pancreaticobiliary malignancies, primary sclerosing cholangitis, and choledocholithiasis. Imaging studies play a key role in ruling out these conditions. The absence of gallstones on imaging excludes choledocholithiasis, while pancreatic or ampullary neoplasms must be excluded through cross sectional imaging, endoscopic ultrasonography, and, in some cases, biopsy. The characteristic tapering of the CBD at the site of PAD compression, as observed in our case, is a hallmark feature of Lemmel's syndrome [14].

The management of Lemmel's syndrome depends on the severity of symptoms and the presence of complications. In uncomplicated cases, conservative treatment is often sufficient. This includes pain management, adequate hydration, and close observation. In this patient, a conservative approach resulted in symptom resolution, including normalization of liver enzyme levels [15].

For patients experiencing recurrent or severe biliary obstruction, endoscopic retrograde cholangiopancreatography (ERCP) is both diagnostic and therapeutic. It can involve sphincterotomy or placement of a CBD stent to relieve obstruction. Surgical interventions, such as diverticulectomy or biliary bypass, may be necessary for patients with recurrent cholangitis, pancreatitis, or chronic obstruction unresponsive to endoscopic treatment. Recent reports have demonstrated successful outcomes with these approaches [6, 16].

Outcomes in Lemmel's syndrome are generally favorable when the condition is diagnosed early and treated appropriately. However, symptom recurrence can occur, particularly in patients with PADs that still have debris and enterolith that do not fully resolve. Regular follow‐up with liver function testing and imaging studies is essential to detect and manage recurrent obstruction or complications at an early stage. Management strategies for Lemmel's syndrome reported in the literature frequently involve invasive interventions such as ERCP with sphincterotomy, biliary stenting, or even surgical diverticulectomy, particularly in patients with recurrent or severe obstruction [10, 16, 17]. In contrast, our patient demonstrated significant clinical and biochemical improvement with conservative measures alone, without requiring endoscopic or surgical therapy. This highlights an important teaching point: in elderly, high‐risk patients, close monitoring and supportive care may be sufficient, underscoring the need for individualized management approaches. In elderly patients, management should be tailored to individual needs, taking into account comorbidities and the patients' ability to tolerate invasive procedures [15, 17, 18].

In summary, it is important to consider Lemmel syndrome as a potential cause of biliary obstruction, especially in elderly patients without gallstones or malignancy. Recognizing this condition and utilizing appropriate imaging and endoscopic techniques can minimize the risk of misdiagnosis and enhance patient outcomes through timely and effective management.

## Author Contributions

**Tinsae Anebo:** conceptualization, data curation, investigation, methodology, supervision, writing – original draft, writing – review and editing. **Michael Negussie:** writing – review and editing. **Phuuwadith Wattanachayakul:** data curation, writing – review and editing. **Karecia Byfield:** writing – review and editing. **Hamza Tahir:** writing – review and editing. **Adrian Zhou:** validation, writing – review and editing. **Ghideon Ezaz:** conceptualization, supervision, validation, writing – review and editing.

## Funding

The authors have nothing to report.

## Consent

Written informed consent was obtained from the patient to publish this report in accordance with the journal's patient consent policy.

## Conflicts of Interest

The authors declare no conflicts of interest.

## Data Availability

The data from the findings of this study were inferred can be obtained from the author upon reasonable request.
